# When Pathologies Collide: The Intersection of Tau and Alpha-Synuclein in Neurodegenerative Diseases

**DOI:** 10.1007/s12035-026-06163-6

**Published:** 2026-09-08

**Authors:** Jenna N. Hunt, Sandhya Kortagere

**Affiliations:** 1 Department of Neurobiology and Anatomy, Drexel University College of Medicine, Philadelphia, PA 19129 USA; 2 Department of Microbiology and Immunology, Drexel University College of Medicine, Philadelphia, PA 19129 USA

**Keywords:** Neurodegenerative diseases, Parkinson’s disease, Tau, Synuclein, Synucleinopathy, Mixed proteinopathies

## Abstract

Neurodegenerative diseases, including Alzheimer’s disease (AD) and Parkinson’s disease (PD), are becoming increasingly prevalent in today’s aging population and with significant cost to society. While these diseases were traditionally defined by distinct protein aggregates, namely, tau tangles and amyloid-β (Aβ) plaques in AD and α-synuclein (α-syn) inclusions in PD, substantial evidence reveals frequent mixed pathologies, with tau, α-syn, and Aβ aggregates coexisting in patients. This copathology complicates understanding disease mechanisms, classification, and progression, highlighting the need to examine mixed pathologies in neurodegenerative diseases to develop disease-modifying therapies. The presence of mixed proteinopathies suggests shared or converging pathological mechanisms, including synergistic aggregation, enhanced seeding capabilities, and shared protein–protein interactions. Understanding these molecular mechanisms is essential for identifying disease modifiers and refining experimental models. This review explores the pathogenic mechanisms of α-syn and tau individually, followed by exploration of their molecular interactions and potential mechanisms of coaggregation and exacerbation of pathology. By recognizing the intersection of these pathologies, further research can refine disease classifications for neurodegenerative diseases.

## Introduction

Neurodegenerative diseases represent one of the greatest medical and societal challenges of the twenty-first century. Alzheimer’s disease (AD) is the most common neurodegenerative disease, affecting about 52 million people globally [1]. Parkinson’s disease (PD) is the second most common neurodegenerative disorder, with over 6 million adults living with PD globally as of 2023 [2]. With the dramatic increase in life expectancy around the globe, this number is expected to grow to between 12 and 17 million people by 2040 with PD [3] and to 153 million in 2050 with AD [1]. Beyond prevalence, these diseases collectively account for billions of dollars in healthcare costs annually, strain caregiving networks, and drastically reduce quality of life for patients and families alike [4]. Despite this overwhelming burden on individuals and society and decades of research, disease-modifying therapies either have not been developed or are variable in their efficacy [5, 6]. Therefore, it is essential to gain a deeper understanding of the neurobiological mechanisms underlying neurodegenerative diseases so better therapies can be developed.

Traditionally, neurodegenerative diseases were conceptualized as distinct pathological entities, each defined by a single protein aggregate. AD is classically characterized by the accumulation of extracellular amyloid-β (Aβ) plaques and intracellular neurofibrillary tangles composed of hyperphosphorylated tau [7, 8], whereas PD is defined by intraneuronal inclusions known as Lewy bodies, largely composed of aggregated α-synuclein (α-syn) [9, 10]. This “one protein-one disease” paradigm provided a useful framework for early classification of neuropathology and therapeutic targeting. However, accumulating evidence has begun to complicate this neat separation. Analysis of postmortem brain samples has revealed that mixed pathologies are more common than previously thought: many patients diagnosed clinically with AD or PD harbor not only their “primary” hallmarks but also additional protein aggregates, including tau, α-syn, TDP-43, or combinations thereof [11–14]. In fact, copathology with tau and α-syn is highly prevalent, with 30% of patients with Lewy body disorders showing high levels of neurofibrillary tau tangles and reduced interval between onset of motor and dementia symptoms [15]. Similarly, nearly half of sporadic AD patients were found to have α-syn-positive inclusions, with a third having α-syn inclusions in all four key brain regions studied [16]. Furthermore, a subset of AD patients exhibit motor symptoms reminiscent of PD, suggesting dopaminergic system involvement and possible α-syn aggregation [17].

The recognition of overlapping pathologies has had important clinical consequences. Patients with PD frequently develop cognitive impairment and dementia, conditions historically attributed to AD but increasingly understood to involve tau and Aβ co-pathology in the context of α-syn inclusions [12, 15, 18]. Lewy body dementia and Parkinson’s disease dementia further exemplify this diagnostic overlap, with mixed α-syn, tau, and Aβ pathology contributing to the clinical heterogeneity [11, 18, 19]. This mixed pathology complicates not only diagnosis and prognosis but also the design and interpretation of clinical trials. Therapeutics aimed at reducing Aβ burden, for example, may fail to demonstrate efficacy in part because tau and α-syn pathology simultaneously drive neurodegeneration [19]. Similarly, α-syn-targeted therapies may be undermined by concurrent tau-driven dysfunction.

Understanding why and how these proteinopathies coexist is therefore essential. Several hypotheses have emerged: (1) aggregation of multiple proteins converges on shared molecular pathways, such as synaptic dysfunction [20]; (2) aggregated proteins may physically interact and coaggregate, thereby amplifying each other’s pathology [21]; and (3) common cellular stressors, such as mitochondrial dysfunction, oxidative stress, impaired proteostasis, and neuroinflammation, create an environment conducive for multiple proteinopathies [22]. Most likely, it is a combination of all these three mechanisms that needs to be accounted for in understanding disease pathology.

**Fig. 1:**
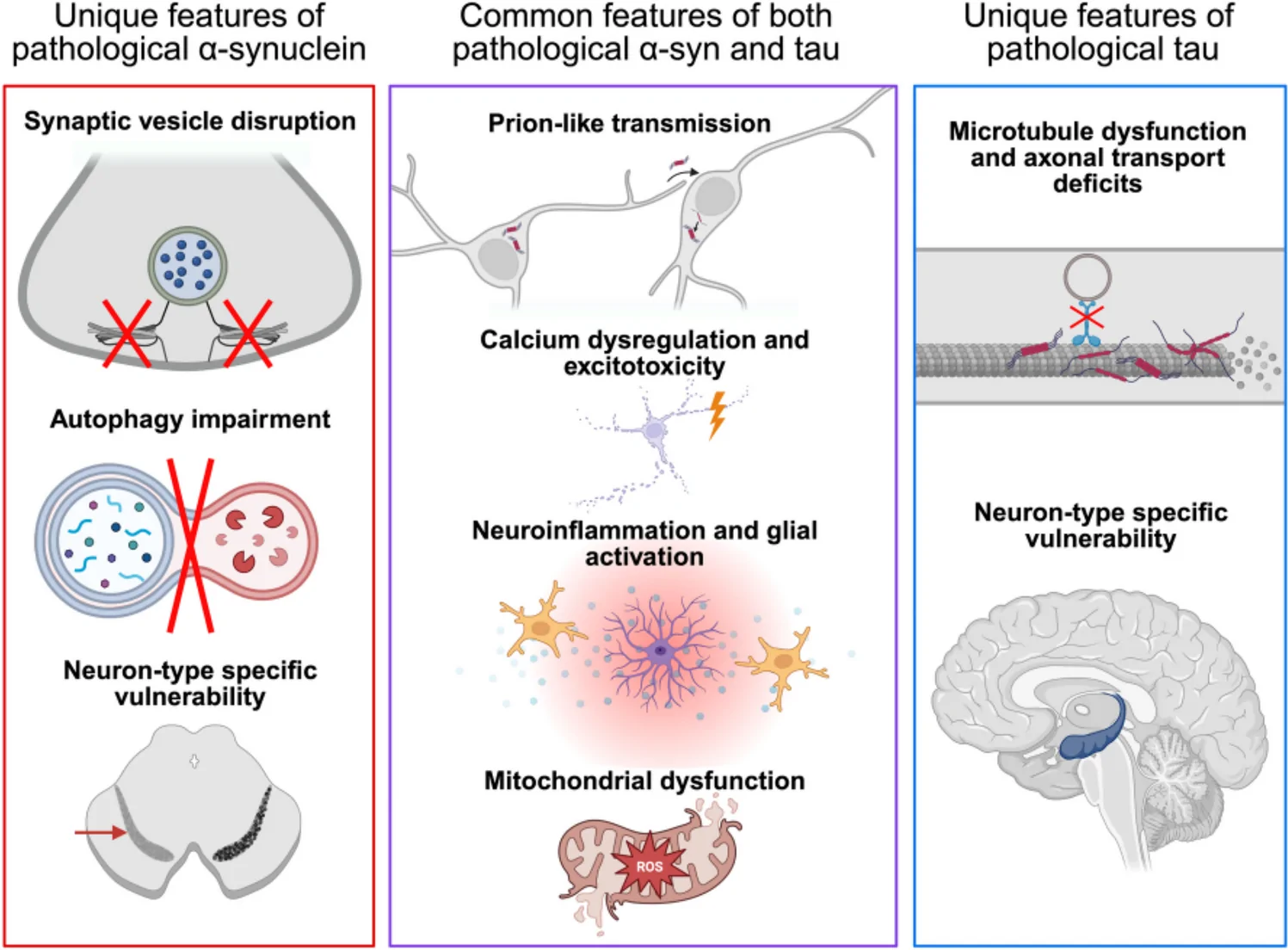
Unique and shared features of pathological α-synuclein and tau*.* Schematic overview comparing disease-associated mechanisms driven by pathological α-synuclein and tau. The left column highlights processes unique to pathological α-synuclein, including disruption of synaptic vesicle dynamics, impairment of autophagic pathways, and selective vulnerability of dopaminergic neurons in the substantia nigra. The central column depicts pathological processes common to both α-synuclein and tau: prion-like propagation between cells, calcium dysregulation and excitotoxicity, neuroinflammation with glial activation, and mitochondrial dysfunction. The right column highlights features unique to pathological tau: microtubule destabilization, deficits in axonal transport, and selective vulnerability of neurons in the hippocampus and entorhinal cortex

This review seeks to synthesize current knowledge on the interplay between tau and α-syn in neurodegenerative diseases. We begin by examining the individual pathogenic features of each protein, emphasizing how they disrupt cellular homeostasis through distinct but often overlapping pathways (Fig. 1). We then turn to evidence of their molecular interactions, including coaggregation and posttranslational modifications. Finally, we explore how these interactions manifest in experimental models and human pathology, consider the implications for diagnosis and therapy, and highlight future directions in the study of mixed proteinopathies. By reframing AD, PD, and other neurodegenerative disorders as multiprotein diseases, we posit the necessity of new preclinical models and therapeutic strategies that embrace their inherent complexity and heterogeneity.

## Unique Features of α-syn and Tau Pathology

###  Unique Features of α-syn Pathology

**Fig. 2:**
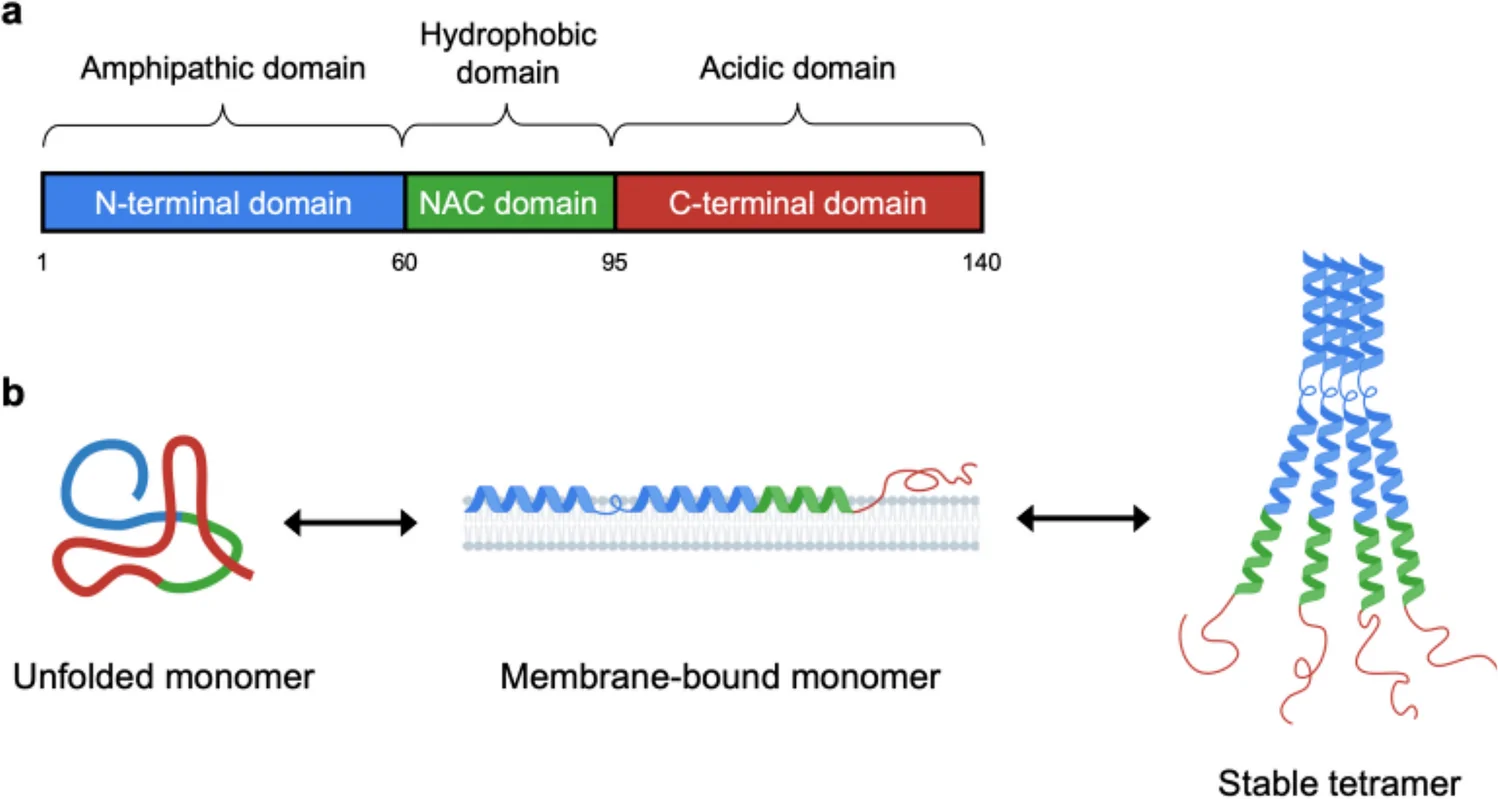
Alpha synuclein protein structure and physiological conformations. **a** Schematic representation of the three major domains of α-synuclein: the N-terminal amphipathic region, which mediates lipid and membrane interactions; the central hydrophobic non-amyloid-β component (NAC) region, critical for aggregation propensity. The acidic, proline-rich C-terminal domain contributes to protein–protein interactions and regulation of aggregation. **b** Physiological conformations of α-synuclein. In the cytosol, α-synuclein can exist as an intrinsically disordered, unfolded monomer. Upon association with lipid membranes, the N-terminal region adopts an α-helical structure. α-synuclein has been proposed to form a soluble, α-helical, aggregation-resistant tetramer under physiological conditions. Together, these conformations highlight the structural plasticity of α-synuclein and its ability to dynamically convert between functional states depending on cellular context

α-syn was first discovered as a small (~ 14 kDa) protein primarily localized to the presynaptic terminal of neurons [10, 23]. While the full extent of its physiological function is not completely understood, it is thought to regulate synaptic vesicle trafficking [24, 25] and dopaminergic neurotransmission [26, 27]. α-syn is an intrinsically disordered protein, meaning that it possesses a high degree of conformational flexibility in its physiological monomeric form (Fig. 2) [28]. While α-syn generally exists as monomers [29] or stable tetramers [30], it has a high propensity to form more complex three-dimensional structures such as oligomers and protofibrils [31]. Many studies have investigated the factors that contribute to the oligomerization of α-syn, such as genetic mutations [32] and posttranslational modifications [33]. These aggregated species have been shown to be toxic to neurons and have been hypothesized to contribute to the pathophysiology of PD [34, 35].

#### Synaptic Vesicle Disruption

In line with its physiological role at the synapse, aggregated α-syn can induce synaptic dysfunction, acting as one of the earliest pathogenic events in PD and other synucleinopathies [20]. Under normal conditions, α-syn regulates synaptic vesicle trafficking and neurotransmitter release at presynaptic terminals [24, 25]. However, when aggregated α-syn accumulates, it disrupts vesicle dynamics and initiates synaptotoxic cascades that precede overt neurodegeneration [36–40].

Pathological α-syn species, particularly oligomers and protofibrils, interfere with both vesicle recycling and neurotransmitter release. Overexpression and/or aggregation of α-syn has been shown to alter distribution of vesicles [40], reduce vesicle pool size [38], enlarge vesicles [36], and sequester or mislocalize key SNARE proteins such as SNAP25, syntaxin-1, and synaptobrevin-2 [37, 39], thereby impairing the fusion machinery required for neurotransmitter release. Specifically, α-syn oligomers have been shown to interact with the synaptobrevin-2 of the SNARE complex in order to inhibit membrane docking of vesicles [37]. In accordance with the in vitro data, multiple studies from both human postmortem patients and rodent PD models have shown a decrease in SNARE and other synaptic proteins such as synapsin 1/2, CSPα, VAMP-2, SNAP-25, and syntaxin [36, 38, 39, 41]. Ultimately, this dysfunction precedes and culminates in neuronal loss in late-stage PD [41, 42].

#### Autophagy Impairment

Under physiological conditions, misfolded wild-type α-syn is primarily degraded by autophagy, particularly chaperone-mediated autophagy (CMA), a selective process involving recognition by HSC70 and translocation through the lysosomal receptor LAMP2A [43, 44]. Pathological α-syn mutants such as A53T and A30P, however, bind to LAMP2A but fail to translocate, thereby preventing their own degradation and that of other substrates [45]. Additionally, levels of LAMP2A have been shown to correlate directly with levels of CMA activity [46, 47]. This is consistent with human post-mortem PD brain samples, where LAMP2A and HSC70 were significantly reduced in the substantia nigra and amygdala [48]. This dysfunction in CMA has been shown to provoke a compensatory induction of macroautophagy, a less selective process involving autophagosome formation and fusion with the lysosome [49]. Instead of helping degrade the misfolded α-syn, this response often accelerates cell death as the lysosomes exceed their capacity for degradation [49]. This has been shown through impaired autophagosome–lysosome fusion through the decrease of the v-SNARE protein SNAP29 [50] and a decrease of the autophagosomal membrane-associated protein LC3 [43].

Furthermore, both in vivo and in vitro studies have highlighted the therapeutic potential of restoring autophagy and the causative role of autophagy dysfunction in neuronal death. In an α-syn-overexpressing neuronal cell line, coexpressing the positive autophagy regulator beclin 1 reduced accumulation of α-syn and reduced neuritic pathology, while in vivo, beclin 1 injections reduced alterations in the autophagy pathway and ameliorated the synaptic and dendritic pathology [51]. Similarly, overexpressing SNAP29 along with α-syn in cultured human neurons reversed the α-syn-induced changes in autophagy and reduced dopaminergic cell death [50]. Taken together, these data suggest the impairment of autophagy from both the chaperone-mediated mechanism and macroautophagy as a result of mutated or overexpression of α-syn.

#### Neuron Type–Specific Vulnerability

Although α-syn aggregation and toxicity can affect multiple neuronal populations, the disproportionate susceptibility of dopaminergic neurons of the substantia nigra pars compacta (SNpc) accounts for the characteristic motor deficits seen in PD [52]. An approximate loss of between 30 and 50% of dopaminergic neurons in the SNpc defines PD [53–55], whereas neuronal loss in other affected regions ranges from 10 to 40% in areas such as the pedunculopontine nucleus and dorsal motor nucleus of the vagus, amygdala, and cortex [56]. This susceptibility of the SNpc arises from a combination of metabolic burden and calcium buffering requirements [52].

Dopaminergic neurons of the SNpc have expansive unmyelinated axonal arbors with hundreds of thousands of synaptic connections per axon [57], placing an immense metabolic burden on these cells to manage high levels of energetic demands, oxidative stress, and proteostasis requirements [58, 59]. Compared to dopaminergic neurons in the ventral tegmental area (VTA), SNpc neurons have higher density of axonal mitochondria and higher rates of basal superoxide production, suggesting a link between elevated oxidative phosphorylation and susceptibility to oxidative stress [60]. Dopamine itself adds to this burden, as cytosolic dopamine can be oxidized into reactive quinones that further damage mitochondria and contribute to lysosomal dysfunction and α-syn accumulation [61]. This toxic cascade involving α-syn and its damaging effects on mitochondria (discussed further in Sect. "Mitochondrial dysfunction") creates a metabolic crisis at least partially unique to SNpc dopaminergic neurons.

Another potential vulnerability of SNpc neurons compared to other dopaminergic neurons lies in their ability to buffer calcium levels inside the cell. Dopamine neurons in the SNpc display pacemaking activity that is a result of L-type-voltage-dependent calcium channel containing a CaV1.3 pore that elevates intracellular calcium levels [62–64]. This is in contrast with VTA dopaminergic neurons, which are more resistant to α-syn pathology and achieve their pacemaking properties through voltage-gated sodium channels [65]. These VTA neurons therefore have lower intracellular calcium levels during pacemaking [66], which may contribute to the relative resistance to excitotoxicity as will be discussed in Sect. "Calcium dysregulation and excitotoxicity". In fact, a mouse model of PD has shown increase in basal firing rates and impaired homeostatic firing regulation of SNpc dopaminergic neurons, while VTA neurons remain unimpaired [67]. Together, these features distinguish SNpc neurons from their more resilient VTA counterparts to drive selective degeneration in PD.

### Unique Features of Tau Pathology

**Fig. 3:**
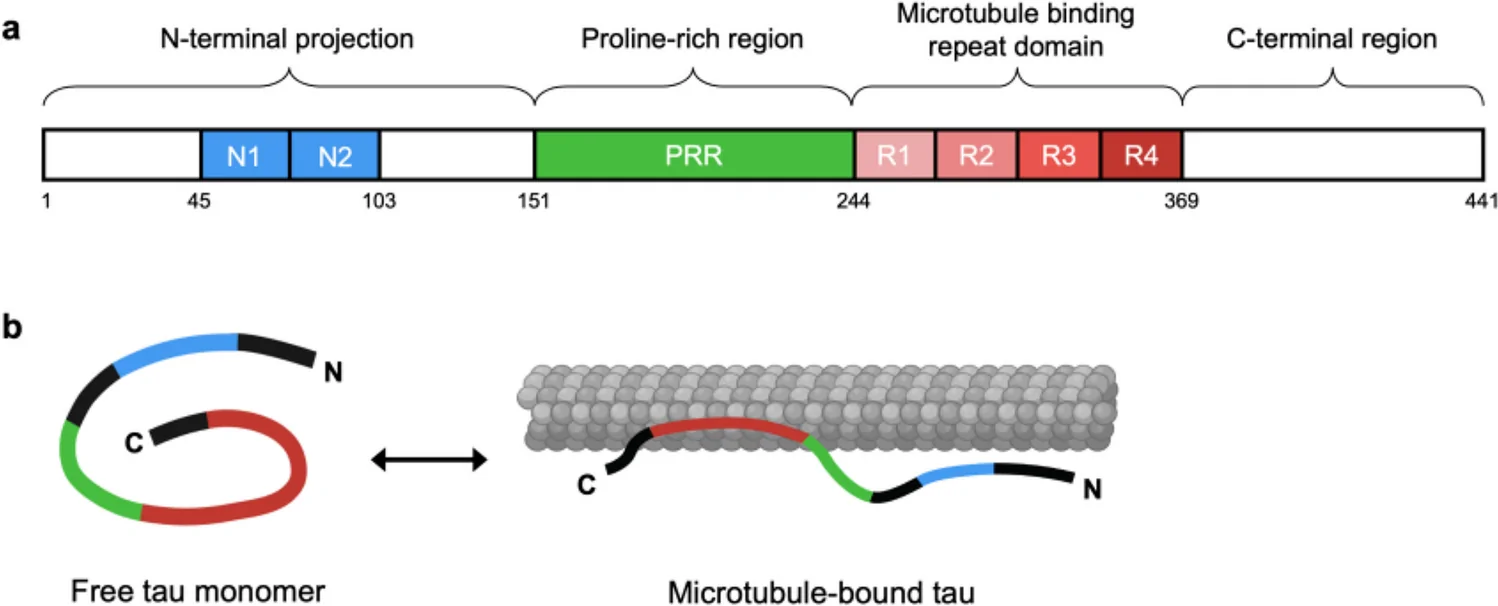
Tau protein structure and physiological conformations. **a** Schematic representation of the tau protein domains shown for the longest human isoform, 2N4R, including the N-terminal projection domain, the proline-rich region, the microtubule-binding repeat domain containing four repeats, and the C-terminal region. **b** Physiological conformations of tau. In solution, tau adopts a dynamic “paperclip” monomeric conformation, which exists in equilibrium with microtubule-bound tau, where binding induces structural rearrangements that promote microtubule stabilization. These states highlight the intrinsic disorder and conformational flexibility of tau under physiological conditions

Tau, another intrinsically disordered protein, is a type of microtubule-associated protein that classically is thought to stabilize microtubules [68], but more recently has been shown to allow microtubules to have long labile domains (Fig. 3) [69]. Tau is also implicated in neurodegenerative diseases known as tauopathies, including frontotemporal dementia, progressive supranuclear palsy, and Alzheimer’s disease (as reviewed in [70]). In AD, hyperphosphorylated tau aggregates to form fibrillar neurofibrillary tangles (NFTs) beginning in the entorhinal cortex (EC); in fact, the degree of NFT spread throughout the brain is closely associated with cognitive decline [7]. However, recent research has questioned the notion that NFTs are toxic [71], suggesting that the prefibrillar forms of tau may be more pathogenic [72, 73].

#### Microtubule Dysfunction and Axonal Transport Deficits

Tau’s most distinctive physiological role is regulating the neuronal cytoskeleton. Encoded by the MAPT gene, tau binds to microtubules via its C-terminal repeat domains, promoting their assembly and stability while supporting axonal transport of organelles and vesicles [74, 75]. In tauopathies such as AD, tau becomes hyperphosphorylated at multiple residues, reducing its affinity for microtubules and reducing its stability [76, 77]. This detachment compromises microtubule integrity, leading to fragmentation of the axonal cytoskeleton [77, 78].

It is clear, however, that the axonal transport deficits in tauopathies are not just a result of loss of function of physiological tau. Overexpressing tau can acquire toxic gain-of-function properties by physically obstructing axonal transport, thereby “clogging” axons and slowing axonal trafficking [79, 80]. Among the most affected cargos are mitochondria, whose proper distribution is critical for energy supply and calcium buffering at synapses [81–83]. These defects arise both from tau overexpression, which interferes with kinesin-mediated transport [83], and from hyperphosphorylation at AD-associated AT8 sites [82], which alters microtubule spacing and reduces mitochondrial motility. Notably, suppression of soluble tau, but not fibrillar inclusions, restores mitochondrial distribution, highlighting soluble and posttranslationally modified tau as key mediators of transport deficits and neuronal dysfunction [81]. Taken together, there is a clear link between phosphorylation of tau and its ability to disrupt axonal transport through loss of function and/or gain of function mechanisms.

#### Neuron Type–Specific Vulnerability

Tau pathology in AD follows a selective pattern, beginning in the EC and hippocampus, while regions such as primary sensory cortices remain relatively resistant [84]. This selective vulnerability reflects both network-mediated spread and intrinsic cellular properties. Models of tau propagation show that pathology often tracks along anatomical connectivity [85], but residual variance highlights contributions from genetic and cell type–specific factors [86, 87].

At the cellular level, transcriptomic and histological analyses have identified specific neuronal subtypes that are disproportionately affected by tau. EC layer II neurons are among the most vulnerable [88], in part due to dysregulation of activity-dependent transcription mediated by factors such as the oncogene DEK, leading to altered excitability, microglial reactivity, and somatodendritic tau accumulation [89]. Single-nucleus sequencing has revealed additional vulnerable populations characterized by dysregulation of apoptosis pathways, vesicle trafficking, and cytoskeletal remodeling [87]. Further gene pathways conferring risk/resilience for tau pathology from imaging-based and histological studies include Aβ formation, cytokine production, protein folding, synaptic organization, and calcium homeostasis [88, 90].

On a cell type–specific level, large-scale mapping further indicates that hippocampal glutamatergic neurons are strongly associated with tau deposition, whereas cortical glutamatergic and GABAergic neurons, as well as oligodendrocytes, appear relatively resilient [91]. These findings support a model in which tau toxicity arises through an interplay between network connectivity, genetic risk, and intrinsic neuronal identity.

## Converging Features of α-syn and Tau Toxicity

### Prion-Like Cell-to-Cell Transmission

One important similarity between pathological α-syn and tau is their ability to spread across interconnected networks of neurons, acting as a key driver of PD and AD progression, respectively [92–94]. Cell culture studies provided the first direct evidence of this mechanism of α-syn propagation. Exogenous recombinant α-syn fibrils, even in small amounts, seed aggregation of endogenous protein in cell lines and primary neurons with and without overexpression of α-syn [41, 95, 96]. Once internalized, fibrils transform soluble α-syn into insoluble, hyperphosphorylated, and ubiquitinated inclusions resembling Lewy bodies, leading to loss of synaptic proteins, altered excitability, and ultimately cell death [41]. Mouse models extend these findings in vivo: intracerebral inoculation of synthetic fibrils or diseased brain homogenates induces widespread pathology, accelerating both Lewy body formation and neurological decline [34, 97]. A single striatal injection of fibrils in wild-type mice is sufficient to trigger progressive nigral dopamine neuron loss, dopamine depletion, and motor deficits, establishing a mechanistic link between prion-like propagation and PD phenotypes [34]. Extending these observations, duodenal wall injections in α-syn-overexpressing rats revealed progressive spread from the enteric nervous system through sympathetic and parasympathetic circuits into the brainstem, heart, and locus coeruleus, where pathology persisted for months [98].

Evidence of α-syn transmission has also been seen in humans through cell transplantation studies in human PD patients [99, 100]. Fetal mesencephalic neurons that have been transplanted into subjects with PD have shown long-term health and integration with host tissue for up to 14 [99] or 16 years [100]. In these subjects, Lewy bodies and Lewy neurites were observed in the grafted dopaminergic neurons that were positive for phosphorylated α-syn and ubiquitin [100] and surrounded by activated microglia [99]. These data suggest a prion-like spread between host-derived pathological α-syn and the initially nonpathological graft cells.

Moreover, pathology is not restricted to the central nervous system (CNS) but can also be initiated from the periphery. In α-syn transgenic mouse models, intramuscular injection of fibrils into the hind limb induces rapid and predictable CNS pathology, accompanied by astrogliosis, microgliosis, and motor impairments [101]. Intraperitoneal and intravenous routes also show spreading with fibril injections triggering CNS α-syn aggregation and, in some cases, paralysis [101, 102]. Together, these studies highlight the prion-like capacity of α-syn to invade the CNS from multiple peripheral entry points, reinforcing the idea that PD progresses through cell-to-cell transmission of α-syn.

Tau also demonstrates a similar pattern of prion-like propagation. In cell lines and in primary neuron cultures, internalized tau fibrils trigger fibrillization of intracellular tau and can be taken up by neighboring cells [103, 104]. These in vitro findings demonstrate that tau propagation contains two important prion-like properties, templated misfolding and self-perpetuating spread. In vivo studies extend these findings to the intact brain. Injection of tau-containing brain extracts into transgenic mice expressing wild-type human tau induces tau aggregation at the site of inoculation and in synaptically connected regions, confirming that tau can propagate along neural circuits [85]. When mutant tau expression is restricted to the EC, pathology spreads sequentially to the dentate gyrus, CA1, and cingulate cortex, recapitulating the stereotyped anatomical progression observed in human disease [105, 106]. This spread is accompanied by synaptic degeneration and neuronal loss in affected regions, supporting a causal link between tau propagation and neurodegeneration [105].

Another important characteristic of prions is the presence of distinct strains that display stable structural phenotypes [107]. In fact, distinct tau strains do maintain their conformational properties and induce reproducible patterns of pathology when transferred between mice, demonstrating that tau aggregates can preserve structural information that dictates the distribution and severity of disease [107]. Moreover, different conformations of tau derived from AD patient brains induce aggregation at distinct rates and with unique structural profiles; this conformation specificity being an important prion-like characteristic [108]. Collectively, in vitro and in vivo studies provide compelling evidence that tau aggregates propagate through neural networks, converting normal tau into pathological fibrils and spreading across the brain.

### Calcium Dysregulation and Excitotoxicity

Another shared mechanism between pathological tau and α-syn is their ability to cause glutamate and calcium dysregulation, leading to excitotoxicity. Pathological α-syn disrupts neuronal calcium homeostasis, driving excitotoxicity and synaptic failure. In primary neurons, oligomeric—but not monomeric—α-syn elevates basal intracellular calcium levels [109]. These oligomers also induce cell death, which was then prevented by blocking extracellular calcium influx [109]. It is hypothesized that this occurs in part due to the formation of pore-like structures, allowing uncontrolled ion entry [109, 110]. In hippocampal cultures, extracellular α-syn oligomers increase conductance, raise intracellular calcium, and stimulate vesicle release, thereby coupling calcium dysregulation to heightened glutamatergic activity [111].

Calcium-permeable cation channels are similarly dysregulated with α-syn. Aggregated α-syn interacts with and overactivates the calcium ATPase SERCA, leading first to cytosolic calcium depletion and later overload; blocking SERCA restores calcium balance and improves viability in cultured neurons and animal models [112]. The addition of human α-syn to mouse primary neurons also reduces surface expression of NMDA receptor NR1 subunits while simultaneously enhancing the activity of NMDA and calcium-permeable AMPA receptors, a finding that was recapitulated in α-syn transgenic mice [113]. This dual role of SERCA overactivation and glutamate receptor dysregulation results in both cell death and synaptic dysfunction, both of which significantly contribute to PD pathology.

Glia further amplify these excitotoxic effects. α-syn aggregates activate microglia through TLR2 and P2X7 receptors, triggering glutamate release via the system Xc⁻ antiporter [114]. Oligomers also stimulate astrocytic glutamate release and activate extrasynaptic NMDA receptors, contributing to dendritic spine loss [115]. In vivo, striatal α-syn injections impair NMDA receptor function in spiny projection neurons and induce tonic glutamate release and extrasynaptic NMDAR-mediated synaptic loss [115]. Together, these findings indicate that α-syn aggregates destabilize calcium regulation at multiple levels—membrane permeability, intracellular buffering, receptor trafficking, and glial glutamate signaling—ultimately tipping neurons into excitotoxic stress.

Tau aggregation and aggregation-prone mutations also disrupt calcium regulation and glutamatergic signaling, driving excitotoxic stress. In primary neurons, overexpression of tau or its N-terminal fragments induces NMDAR-dependent, caspase-independent cell death, mediated by extrasynaptic NR2B receptors [116]. This signaling cascade includes CREB dephosphorylation, sustained ERK1/2 activation, and calpain overactivation, which cleaves tau into highly toxic fragments, creating a self-reinforcing loop of calcium overload and proteolysis [116]. Extracellular aggregated tau can also directly influence calcium signals by increasing ionic conductance, depolarizing neurons, and activating voltage-gated calcium channels, which also stimulated the production of reactive oxygen species [117]. Similarly, disease-associated tau mutations such as V337M enhance L-type calcium channel activity, resulting in persistent calcium influx and heightened vulnerability to apoptosis [118]. On the other hand, genetic tau deletion or knockdown reduces NMDA receptor-mediated calcium influx and protects against excitotoxic injury [119]. In parallel, astrocytes exposed to extracellular tau oligomers lose glutamate-buffering capacity through GLT-1 downregulation and Na+/K+-ATPase mislocalization, amplifying excitatory imbalance [120].

Mouse models corroborate these excitotoxic mechanisms of pathological tau. Tau transgenic mice expressing a disease-associated tau mutant show hippocampal hyperexcitability, elevated intracellular calcium, and glutamate spillover, particularly in CA3 neurons, where excitotoxic death is mitigated by NR2B antagonists or ceftriaxone-mediated upregulation of astrocytic EAAT2 transporters [121]. Another transgenic mouse line was found to exhibit increased glutamate release and impaired clearance in the hippocampus, leading to elevated extracellular glutamate, synaptic loss, and memory deficits, all of which are improved by inhibiting glutamate release with riluzole [122].

Together, these findings highlight tau as both a driver and amplifier of excitotoxic cascades by destabilizing calcium homeostasis and impairing glutamate regulation.

### Neuroinflammation and Glial Activation

α-syn aggregates provoke strong inflammatory responses across peripheral and central immune systems. In monocytic cultures, α-syn preformed fibrils (PFFs) activate toll-like receptor (TLR) signaling, upregulating NF-κB-dependent inflammatory genes, including *TNF*, *IL1B*, and noncanonical mediators such as *NFKB2*, *CD40*, and *RANK* [123]. This transcriptional shift promotes differentiation toward pro-inflammatory macrophage-like states and amplifies cytokine release [123]. Centrally, iPSC-derived microglia exposed to α-syn fibrils display impaired phagosome maturation, mitochondrial dysfunction, and increased nitric oxide and cytokine production—responses that are further enhanced by IFN-γ costimulation [124]. The formation of the NLRP3 inflammasome in response to α-syn has also shown to be regulated by Fyn kinase and the scavenger receptor CD36 [125]. Similarly, in primary astrocytes, extracellular α-syn activates TLR4-dependent MAPK and NF-κB signaling, inducing iNOS and COX-2 expression, while TLR4 knockout abolishes cytokine induction but not α-syn uptake, indicating distinct pathways for endocytosis and inflammatory transduction [126].

Transmission of α-syn aggregates from neurons to glia also drives pathology. Astrocytes endocytose neuron-released α-syn and form intracellular inclusions that elicit robust transcriptional inflammatory programs marked by chemokine induction and altered metabolic genes [127]. Additionally, PD patient-derived fibrils synergize with TNFα and PGE2 to polarize microglia toward a chronic, neurotoxic state characterized by iron retention, disrupted TCA cycling, and elevated glutamate release via xCT upregulation—thereby promoting dopaminergic neuron death in vitro [128].

Animal studies mirror these early innate immune responses. Intrastriatal α-syn PFF injections in mice induce rapid microglial and astroglial activation within 15 days, preceding α-syn aggregation and dopaminergic loss by months [129]. Rat studies mirror this effect, with MHC-II upregulation and microglial activation peaking months prior to nigral dopaminergic cell loss, with a significant correlation between α-syn inclusion burden and number of microglia expressing MHC-II. This suggests that reactive microglia may contribute to the vulnerability of nigral neurons to degeneration [130]. In a different model, overexpression of mutant A53T α-syn enhanced microgliosis and cytokine production, especially when CX3CR1 signaling is disrupted, highlighting CX3CR1 pathways in modulating inflammatory severity [131].

Collectively, these findings suggest that α-syn is both a trigger and amplifier of glial-mediated inflammation, linking its aggregation and intercellular spread to sustained microglial and astrocytic activation that drives dopaminergic vulnerability and disease progression.

Tau aggregation and inflammation are also tightly linked, with glial activation both responding to and accelerating tau pathology. In primary microglia, tau PFFs activate the TLR2-MyD88-NFκB pathway, inducing proinflammatory cytokine production [132]. Blocking this pathway with a TLR2-interacting domain peptide suppresses gliosis, inflammatory markers, and pathogenic tau accumulation in PS19 mice, improving cognitive function [132]. Tau-induced inflammation also directly increases neuronal tau transcription through NFκB, linking glial activation to the feed-forward escalation of tau pathology [132]. Not only does pathological tau induce an inflammatory response, but the microglial inflammatory response also enhances tau hyperphosphorylation, leading to a positive feedback loop between neuronal tau hyperphosphorylation and microglial activation [133]. In fact, suppressing the microglial inflammatory response by manipulating TREM2 levels ameliorated the tau hyperphosphorylation induced by activated microglia [133].

Astrocytes contribute similarly to this loop. Human iPSC-derived astrocytes internalize brain-derived tau fibrils but fail to degrade them, instead propagating seeding-competent tau through tunneling nanotubes and exocytosis [134]. Subsequently, tau accumulation drives a reactive phenotype characterized by increased GFAP and vimentin expression, secretion of IL-8 and MCP-1, and neurotoxic effects including synaptic and metabolic impairment [134]. Tau also binds astrocytic integrin αV/β1 receptors, activating NFκB signaling and further promoting release of proinflammatory cytokines and neurotoxic factors [135]. Together, these findings show that both microglia and astrocytes act not only as responders but as amplifiers of tau pathology.

In vivo, microglial activation has been shown to either precede or follow tau aggregation. In PS19 mice expressing P301S under the mouse prion protein promoter, microgliosis appears around the same time as phosphorylated tau is detected but after tau tangle formation and synaptic loss, and immunosuppression reduces pathology and extends lifespan [136]. However, neuron-specific overexpression of P301S tau induces early tau aggregation with morphological changes seen in microglia at a later timepoint [137]. This suggests that microglial activation may play a role in the later stages of tau processing, after hyperphosphorylation but prior to tangle formation [136, 137]. Additionally, manipulations of microglial signaling have been shown to affect tau-induced pathology. Loss of the microglial receptor CX3CR1 enhances IL-1 and p38 MAPK signaling, accelerating tau phosphorylation, aggregation, and behavioral decline [138]. Conversely, blocking IL-1β signaling in 3xTg-AD mice mitigates tau pathology, decreases activity of GSK-3β and p38 MAPK, and improves cognition, establishing a causal link between cytokine signaling and tau modification [139].

Collectively, these findings demonstrate that glial activation not only accompanies but also accelerates tau aggregation, propagation, and neurotoxicity through cytokine signaling and establishes neuroinflammation as a central driver of tau-mediated neurodegeneration.

### Mitochondrial Dysfunction

Pathological α-syn also disrupts mitochondrial homeostasis through multiple converging toxic mechanisms that culminate in oxidative stress and bioenergetic failure. In a hypothalamic neuronal cell line, α-syn overexpression induces enlarged mitochondria with abnormal cristae, a 30% decrease in mitochondrial activity, and a compensatory increase in the antioxidant glutathione in response to the α-syn-induced increase in oxidative stress [140]. Other models use extracellular treatment of different α-syn species rather than genetic overexpression. In these models, aggregated α-syn species have been shown to impair complex I-dependent respiration [141] and promote lipid peroxidation and oxidation of ATP synthase subunits [142]. These oxidative events sensitize mitochondria to permeability transition pore opening, triggering swelling, cytochrome c release, and cell death [142]. Similarly, prefibrillar α-syn oligomers disrupt mitochondrial calcium buffering, accelerating swelling, depolarization, and cytochrome c release, especially under conditions of complex I stress [143]. α-syn has also been shown to interfere directly with mitochondrial protein import by binding to TOM20 in vitro, blocking its interaction with TOM22, and leading to deficient respiration, increased ROS production, and loss of mitochondrial membrane potential [144]. This aberrant α-syn-TOM20 interaction has been confirmed in PD postmortem tissue, highlighting its clinical relevance [144]. Taken together, these data suggest severe disruption of mitochondrial form and function with either overexpression or exogenous treatment of aggregated α-syn.

Similar results have been demonstrated in mouse models of PD. Transgenic mice overexpressing A53T α-syn specifically in dopaminergic neurons exhibit early mitochondrial abnormalities, including macroautophagic inclusions containing mitochondrial remnants, complex I inhibition, and lipid peroxidation that precedes cell loss [145]. Deletion of PINK1 or Parkin—both associated with early-onset PD and known players in mitophagy—exacerbates these pathologies, underscoring the importance of mitophagy in counteracting α-syn toxicity [145]. In a similar model, α-syn was also shown to localize to mitochondrial membranes under stress, inhibiting complex I activity and increasing mitophagy [146]. Neuron-type selectivity of α-syn mitochondrial disruption is even seen in mouse models expressing human α-syn in all neurons, where the mitochondrial dysfunction and oxidative damage was mostly localized to the ventral midbrain [147]. In contrast, cortical and striatal regions remained protected despite comparable α-syn accumulation [147]. Together, these studies indicate that α-syn directly targets mitochondria, impairs bioenergetics, and that the high metabolic demand and limited antioxidant capacity of dopaminergic neurons confer selective vulnerability.

In AD and related tauopathies, tau accumulation profoundly disrupts mitochondrial homeostasis, impairing energy production, quality control, and organelle communication. In cellular models, tau localizes to both the outer and inner mitochondrial membranes [148], where caspase-3-cleaved tau selectively disrupts ER-mitochondria contacts and calcium exchange, resulting in altered calcium homeostasis and bioenergetic stress [148, 149]. In addition to altered bioenergetics, mitophagy is also affected by pathological tau; both wild-type and P301L mutant tau inhibit Parkin-dependent mitophagy by sequestering Parkin in the cytosol and preventing its translocation to damaged mitochondria, thereby blocking mitochondrial clearance and promoting the buildup of dysfunctional organelles [150]. Additionally, tau expression in neurons further interferes with mitochondrial dynamics and morphology. Overexpression of full-length or mutant tau enhances mitochondrial fusion through accumulation of mitofusin proteins [151], while stabilization of actin by tau impairs the recruitment of the fission protein DRP1, causing mitochondrial elongation and fragmentation [152]. Hyperphosphorylated tau also directly interacts with DRP1, increasing fission activity observed in both postmortem AD brains and transgenic mouse models [153]. These structural abnormalities are accompanied by reduced ATP production [151], reduced complex I activity [151], and increased oxidative stress [152]. Pathogenic tau mutations induce similar mitochondrial dysfunction, including elevated membrane potential, diminished NADH levels, and excess reactive oxygen species, leading to oxidative damage and neuronal death [117].

In vivo studies further demonstrate that tau accumulation profoundly disrupts mitochondrial structure, dynamics, and function in the brain, leading to synaptic and cognitive decline. In transgenic mouse models, tau associates with both synaptic and nonsynaptic mitochondria, impairing bioenergetics and contributing to loss of excitatory synapses in the hippocampus and cortex [154]. Extending these observations, PS19 tauopathy mice exhibited impairments in mitochondrial oxidative phosphorylation, together with deficits in mitochondrial phosphatidylethanolamine biosynthesis, providing evidence that pathological tau disrupts mitochondrial bioenergetics in vivo [155]. In parallel, abnormal tau weakens mitochondria-associated ER membranes by disrupting the VAPB–PTPIP51 tethering complex, leading to reduced ER–mitochondria coupling and impaired mitochondrial cholesterol metabolism [156].

Together, these findings demonstrate that tau exerts multifaceted toxicity on mitochondria by disrupting fission/fusion dynamics, inhibiting mitophagy, altering calcium signaling and ER–mitochondria communication, and driving oxidative stress. The resulting energy deficits and organelle damage compromise synaptic integrity and neuronal survival, identifying mitochondrial dysfunction as a central downstream consequence of tau pathology.

## Functional Interactions of Tau and α-syn

### Genetic Links Between α-syn and Tau in PD

Genetic studies have consistently implicated both the SNCA and MAPT genes as major contributors to susceptibility to PD, providing one of the strongest links between α-syn and tau pathology. Genome-wide association studies (GWAS) have repeatedly identified the gene encoding for α-syn (SNCA) and the gene encoding for tau (MAPT) as the most robust common genetic risk loci for sporadic PD, highlighting that pathways underlying tauopathies and synucleinopathies are not genetically distinct [157–159]. These findings support the growing recognition that tau contributes to PD pathogenesis despite α-syn aggregation remaining the defining pathological hallmark of the disease.

The principal tau-associated genetic signal in PD arises from the 17q21.31 MAPT inversion haplotype, where the H1 haplotype is consistently associated with increased PD risk, while the H2 haplotype is generally protective [160, 161]. Although the magnitude of risk conferred by individual sub-haplotypes varies across studies and populations, the overall association between H1 and PD has proven reproducible in the literature [159, 162]. Beyond disease risk, the H1 haplotype has been associated with increased MAPT expression in human brain tissue, providing a potential mechanistic link between genetic variation and elevated tau burden [163]. Consequently, MAPT variation may predispose neurons to tau accumulation, creating a cellular environment that facilitates downstream neurodegenerative processes. However, more work must be done to conclusively associate increased tau burden to PD pathogenesis.

Whether MAPT and SNCA interact genetically to influence PD susceptibility remains less clear. Early candidate gene studies suggested synergistic effects between MAPT H1-associated variants and SNCA polymorphisms, reporting substantially greater disease risk and, in some cases, increased susceptibility to cognitive decline among individuals carrying risk alleles at both loci [164]. However, subsequent analyses involving substantially larger cohorts have generally failed to detect significant synergistic or additive interactions, instead indicating that MAPT and SNCA contribute largely independent effects to overall PD susceptibility [159]. Thus, while both loci are unequivocal genetic risk factors, strong evidence for a gene-level interaction has not emerged.

Rather than functioning through a consistent gene–gene interaction, tau may influence PD by modifying disease vulnerability and clinical phenotype. Several studies have associated MAPT variation with cognitive impairment, dementia, and other nonmotor manifestations of PD [165–167], although these associations have not been uniformly replicated [168]. Complementing these findings, network-based analyses have demonstrated that regional MAPT expression correlates more closely with selective neuronal vulnerability and cognitive dysfunction than regional SNCA expression, suggesting that tau-related mechanisms may contribute preferentially to the spread and heterogeneity of neurodegeneration in PD [169].

Collectively, current evidence indicates that the strongest genetic connection between tau and α-syn pathology lies in their shared status as reproducible PD susceptibility loci and their convergence on common pathogenic mechanisms. While MAPT and SNCA appear to influence disease risk largely through independent genetic effects, the MAPT H1 haplotype, combined with extensive experimental evidence for tau–α-syn crosstalk, provides compelling support for tau as an important modifier of PD pathogenesis rather than merely a coincidental copathology.

### Molecular and Biochemical Mechanisms of Interaction

In addition to the biological pathways that α-syn and tau have in common to influence toxicity, accumulating evidence demonstrates that these proteins are capable of direct physical and biochemical interaction. This interaction occurs at multiple levels: transient molecular binding, phosphorylation-dependent modulation, and incorporation into shared fibrillar assemblies. Together, these findings reveal that α-syn and tau are not independent pathological entities but rather members of a shared molecular network whose interactions are intertwined at both a physical level and at the level of biological pathways.

Biochemical analyses have shown that α-syn and tau directly bind through noncovalent electrostatic interactions: α-syn’s acidic C-terminal region engages with the positively charged microtubule-binding domain of tau, facilitating a high-affinity electrostatic association [170, 171]. This physical interaction has also been shown in PD models using cell lines, mice, and rats [172]. In vitro studies using the MPTP model of parkinsonism demonstrate that MPTP increases α-syn levels and enhances tau phosphorylation by kinases such as protein kinase A (PKA) and glycogen synthase kinase-3β (GSK-3β), particularly at Ser262 and Ser356—sites that regulate tau’s ability to bind and stabilize microtubules [173]. Supporting these findings, cellular and animal studies using the MPTP model have identified the formation of heterotrimeric complexes containing α-syn, tau, and GSK-3β [174, 175]. The role of α-syn in the GSK-3β-mediated phosphorylation of tau has been highlighted by the lack of activated GSK-3β in α-syn knockout cells and mice [175]. Similar results were observed in a mouse model overexpressing human α-syn in neurons, where the three proteins were found to be overexpressed and colocalized in large inclusion bodies reminiscent of Lewy bodies [176].

The strength and stability of this interaction are influenced by posttranslational modifications of both proteins. Phosphorylation of α-syn at Ser129, a modification associated with pathological forms of the protein, significantly increases its affinity for tau [170]. Similarly, disease-linked mutations in α-syn (such as A53T) or tau (such as P301L) enhance their ability to co-associate and alter their biochemical behavior in vitro [173, 177]. These findings imply that disease-relevant modifications not only affect the aggregation properties of each protein individually but also govern their binding dynamics.

Beyond simple binding, structural studies provide compelling evidence that α-syn and tau can initiate the fibrillization of each other and physically co-assemble into mixed fibrillar aggregates. Coincubation of tau and α-syn in vitro and in bigenic mice shows that monomeric α-syn and tau can initiate and accelerate each other’s fibrillization, forming hybrid assemblies with features distinct from those of either protein alone [177, 178]. A similar transgenic mouse model has also been shown to have a positive correlation between phosphorylated tau and phosphorylated α-syn in the transgenic mice, suggesting a synergistic interaction between the two proteins [179].

Structural and in *vitro* aggregation studies have also revealed that α-syn and tau can coassemble into mixed fibrillar species. Recombinant experiments and imaging analyses, including optical photothermal infrared microspectroscopy, show that hybrid α-syn/tau fibrils exhibit distinct β-sheet-rich architectures compared to homotypic fibrils of either protein [180]. Furthermore, the tau isoform composition (3R vs. 4R) and the presence of specific α-syn variants influence the structural conformation of these complexes, producing fibril polymorphs with unique biochemical signatures [180]. These hybrid assemblies suggest that α-syn and tau can physically integrate at the level of secondary structure, forming copolymers with intermolecular β-sheet interactions that influence seeding potency.

Despite strong evidence for direct molecular interactions between α-syn and tau, the spatial organization of these pathologies in vivo remains an area of active investigation. Human postmortem studies and cellular models have demonstrated colocalization of phosphorylated α-syn and tau within individual neurons, neurites, synaptic terminals, and Lewy body-associated inclusions, supporting the existence of direct intracellular interactions [181, 182]. However, accumulating evidence suggests that synergistic pathology does not require both proteins to aggregate within the same cell. Instead, α-syn and tau can propagate through interconnected neural circuits, with α-syn pathology often enhancing the accumulation and spread of tau more strongly than tau promoting α-syn pathology, consistent with a regional cross-seeding model [183, 184]. Recent work further indicates that the formation of true hybrid α-syn + tau coaggregates is context- and seed-dependent rather than universal, with some aggregate conformations producing extensive colocalization while others generating predominantly adjacent but distinct pathologies [180].

Taken together, tau and α-syn engage in a dynamic biochemical relationship, reciprocally influencing each other’s posttranslational modification, localization, conformational state, and propagation, thereby linking their molecular behaviors to disease-relevant processes. Although tau and α-syn copathology can occur within the same cell in human brains and experimental models, the broader literature supports a model in which regional, circuit-level interaction drives the propagation of pathology, with intracellular coaggregates arising only in specific cellular and pathological contexts.

### Cellular and In Vivo Consequences of Copathology

Growing evidence demonstrates that tau and α-syn do not act as independent pathogenic entities but rather engage in a reciprocal relationship that amplifies aggregation and toxicity. Studies across experimental models—from cell culture to transgenic mice—consistently reveal that when tau and α-syn coexist, both proteins exhibit heightened pathological accumulation and increased neurotoxicity.

In vitro studies provide the first direct evidence of physical and pathological interaction between tau and α-syn. Cell culture models have shown that tau colocalizes with α-syn aggregates and directly alters α-syn aggregation patterns [185]. Overexpression of tau leads to smaller, more numerous α-syn inclusions and increases the levels of insoluble α-syn species [185]. These changes are accompanied by elevated secretion of α-syn and increased cytotoxicity, indicating that tau expression not only reshapes α-syn aggregation dynamics but also enhances its pathological spread and cellular toxicity [185]. Complementary biochemical studies have shown that short hydrophobic α-syn peptides can independently drive the aggregation of full-length tau in the absence of heparin, producing fibrillar and amorphous aggregates with distinct conformational changes [186]. When these aggregates are applied to cultured neuronal cells, they induce morphological abnormalities and cell death, further supporting a direct cytotoxic outcome of tau–α-syn interactions [186]. PD-associated α-syn mutants have also been shown to destabilize microtubules with higher potency when coexpressed with tau compared to α-syn alone [173]. Similarly, a tau-modified α-syn fibril model in mice showed enhanced seeding activity of the fibrils compared to either protein alone as well as increased mitochondrial dysfunction, synaptic impairment, and neurotoxicity [187]. Together, these in vitro findings indicate that tau and α-syn form a mutually reinforcing relationship that converts normally regulated protein behavior into a toxic aggregation cascade.

In vivo studies extend this evidence, showing that the co-occurrence of tau and α-syn pathologies enhances molecular markers of disease across multiple models. In transgenic mice overexpressing human α-syn under the PDGF promoter, significant accumulation of hyperphosphorylated tau was observed in the striatum alongside aggregated α-syn and activated GSK-3β [176]. This was accompanied by cytoskeletal disruption and brain atrophy, suggesting that α-syn expression accelerates tauopathy and structural degeneration [176]. Consistent results were seen in mixed pathology models combining Alzheimer’s- and Lewy body-related features: introduction of human α-syn into 3xTg-AD mice intensified the accumulation of Aβ, tau, and α-syn and was associated with more pronounced neurodegeneration [188]. Similar findings were seen with double transgenic mice expressing both P301S tau and A53T α-syn, which experienced increased phosphorylated tau in the hippocampus and cognitive impairments in the double transgenic mice compared to tau-only mice [189]. These results extend to “gut-first” models of PD, where transgenic mice expressing gut-inducible SYN103 and TAU368 had increased spreading of both proteins and more widespread neuron loss compared to single transgenic mice of either protein alone [190]. This enhanced pathology in mixed models has also been reported using the fibril seeding model, where co-injection of α-syn PFFs and AD-derived tau seeds was found to increase tau spreading and aggregation both in vitro and in vivo [183]. Notably, the absence of endogenous α-syn in mice reduced the accumulation and spreading of tau pathology, indicating that α-syn may be a strong driver of this pathological interplay [183]. These findings underscore that tau–α-syn interactions converge on enhanced protein deposition and neurodegenerative pathology in multiple models of α-syn–tau copathology.

The cellular and molecular consequences of copathology translate into pronounced behavioral and physiological impairments. First, evidence of tau as a mediator in α-syn-overexpressing models can be seen in vivo. In α-syn transgenic mice, overexpression of α-syn leads to mislocalization of tau to postsynaptic spines, resulting in postsynaptic dysfunction and memory deficits, implicating tauopathy as a downstream amplifier of α-syn-driven neurodegeneration [191]. Importantly, removal of endogenous mouse tau completely ameliorated this synaptic dysfunction and cognitive deficits, highlighting tau as a mediator of this α-syn-induced synaptic dysfunction [192]. Subsequent work using α-syn PFFs confirmed that tau deletion delayed motor impairment, reduced neurodegeneration, and prolonged survival, suggesting tau acts downstream of α-syn aggregation to worsen disease progression [193]. A complementary study using tau oligomer-specific antibody treatment in α-syn A53T mice further supports this interaction: depletion of tau oligomers not only reduced toxic tau species but also protected against α-syn-induced cognitive and motor decline [194].

This interaction can also be seen in models coexpressing α-syn and tau. In a model with mutant human α-syn in 3xTg-AD mice, the addition of the α-syn transgene accelerated cognitive decline and amplified α-syn, tau, and Aβ pathology, paralleling the aggressive progression observed in human cases of mixed pathology [188]. Furthermore, mice injected with tau-modified α-syn fibrils exhibited more severe α-syn pathology, mitochondrial dysfunction, and both motor and cognitive impairments than those injected with unmodified α-syn fibrils, whereas tau knockout attenuated these effects [187]. Together, these behavioral findings converge on a consistent pattern: co-occurrence of α-syn and tau pathologies leads to accelerated and amplified cognitive and motor decline, while disruption of either component—particularly tau—confers significant functional protection. This growing body of evidence emphasizes that behavioral outcomes in α-synucleinopathies and tauopathies are not merely additive but reflect a pathological synergy that drives more severe neurological impairment.

## Sex Differences in α-syn and Tau Pathology in PD

Clinical and preclinical studies consistently demonstrate stronger sex differences for α-syn pathology than for tau pathology [195]. In humans, men exhibit greater PD vulnerability and several sex-dependent α-syn biomarker profiles. Plasma α-syn declines during advanced disease only in men and correlates with cognitive impairment, hallucinations, and sleep dysfunction exclusively in male patients [196]. Furthermore, testosterone levels inversely correlate with CSF α-syn levels in men, while proinflammatory immune signatures are enriched in male PD patients and associate with greater central synucleinopathy, suggesting that hormonal and immune mechanisms contribute to sex-dependent disease progression [197, 198].

Animal models similarly demonstrate greater male susceptibility to α-syn pathology. In A53T transgenic and SNCA-overexpression mice, males develop earlier axonal degeneration, motor impairments, and higher microglial activation than females, indicating accelerated disease progression [199, 200]. Notably, the loss of estrous cycling, aging, and immune alterations diminish this protection, resulting in an eventual convergence of disease burden in aged mice of both sexes [200, 201]. Importantly, sex differences extend beyond neurons, as males generally exhibit earlier proinflammatory immune responses, whereas females display more dynamic or regulatory inflammatory profiles that alter α-syn pathology and disease trajectory in mice [201, 202].

Compared with α-syn, evidence for sex differences in tau pathology within PD remains more limited but suggests that sex modifies the relationship between Lewy pathology and tau accumulation rather than affecting tau pathology independently. In PD, circulating sex hormones correlate with CSF total tau and phosphorylated tau levels, supporting hormonal regulation of mixed proteinopathies [197]. Similarly, neuropathological studies demonstrate that although men exhibit greater Lewy body pathology overall, women with Lewy body disease accumulate more tau than men after controlling for α-syn pathology, and in women with earlier menopause, further strengthening this association [203]. Consistent with these observations, a longitudinal α-syn seed amplification study reported that women positive for α-syn pathology experienced the most rapid tau accumulation, although this cohort was not exclusively composed of PD patients [204].

Collectively, these findings emphasize that sex should be considered as a variable affecting pathology, neuroinflammatory responses, and biomarkers rather than simply a covariate in PD research. Experimental design should therefore include both sexes, multiple age groups, and longitudinal analyses, as females may exhibit delayed symptom onset despite early pathological changes [200, 205]. Furthermore, because α-syn propagation differs between protein overexpression and preformed fibril models, sex-specific effects may vary depending on the experimental paradigm employed [206]. While evidence for sex-dependent α-syn pathology is currently more robust than for tau, the available data indicate that biological sex influences the development of both α-syn and tau pathology and should therefore be incorporated into future mechanistic studies.

## Other Pathogenic Proteins in Neurodegenerative Diseases

**Fig. 4:**
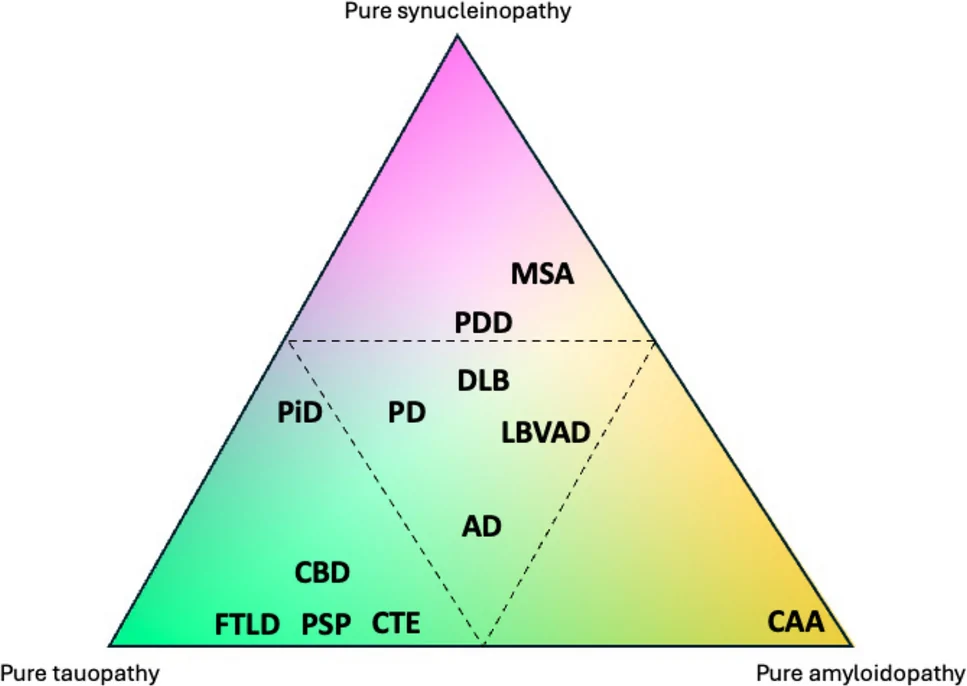
Spectrum of neurodegenerative diseases involving α-syn, amyloid-β, and tau. Schematic illustrating the continuum of neurodegenerative diseases defined by the relative contributions of pathological α-syn, tau, and amyloid-β. Diseases are positioned within the triangle according to the extent of mixed versus predominant protein involvement, with proximity to a vertex indicating a more “pure” single-protein pathology. Disorders shown include multiple system atrophy (MSA), Parkinson’s disease (PD), Parkinson’s disease dementia (PDD), dementia with Lewy bodies (DLB), Lewy body variant of Alzheimer’s disease (LBVAD), Alzheimer’s disease (AD), corticobasal degeneration (CBD), frontotemporal lobar degeneration (FTLD), progressive supranuclear palsy (PSP), Pick’s disease (PiD), chronic traumatic encephalopathy (CTE), and cerebral amyloid angiopathy (CAA)

While α-synuclein and tau represent the primary protein aggregates implicated in PD pathogenesis, the broader field of neurodegeneration has increasingly recognized the contribution of multiple aggregation-prone proteins across different disorders. Two notable examples are amyloid-β (Aβ) and transactive response DNA-binding protein 43 kDa (TDP-43), both of which are associated with distinct neurodegenerative diseases but have also been observed in subsets of PD patients [207]. The most prevalent protein aggregates (α-syn, tau, and Aβ) and the diseases they are implicated in are shown in Fig. 4.

Aβ is a peptide generated through the proteolytic processing of amyloid precursor protein (Chen 2017). Although its normal physiological functions are not fully understood, Aβ is thought to play roles in synaptic plasticity and memory [208]. Pathological accumulation of Aβ into extracellular plaques is a defining hallmark of Alzheimer's disease and is strongly associated with cell death [121]. In PD, Aβ pathology is not considered a primary disease driver; however, cortical Aβ deposition is frequently observed in patients with Parkinson's disease dementia and dementia with Lewy bodies [209, 210]. The presence of Aβ pathology has been associated with greater cognitive decline in PD patients and may contribute to the heterogeneity of clinical symptoms observed across synucleinopathies [207, 210]. In fact, recent amyloid PET studies reveal increased prevalence in dementia with Lewy body patients compared to PD patients [211].

TAR-DNA-binding protein-43 (TDP-43) is a predominantly nuclear RNA- and DNA-binding protein involved in RNA processing, transcriptional regulation, and maintenance of cellular homeostasis. Under pathological conditions, TDP-43 can become mislocalized to the cytoplasm, where it forms ubiquitinated aggregates [212]. TDP-43 pathology is most strongly associated with amyotrophic lateral sclerosis and frontotemporal dementia, in which it forms cytoplasmic inclusions in the spinal cord and cortex, respectively [213]. Although less common in PD than α-synuclein or tau pathology, TDP-43 inclusions have been identified in a subset of PD cases (10–20%), particularly in patients with cognitive impairment or dementia [214]. TDP-43 has also been shown to interact with other aggregated proteins such as tau, α-syn, huntingtin, and ataxin-2 and promote aggregation and/or worsened disease progression [183]. This abundant crosstalk between these proteins further highlights the complexity and overlap of proteinopathies across neurodegenerative disorders.

The presence of Aβ and TDP-43 pathology in some PD patients highlights the broader complexity of neurodegenerative disease. Rather than being defined by a single pathogenic protein, most neurodegenerative disorders exhibit various combinations of pathogenic proteins that contribute to disease progression.

## Limitations and Conclusions

### Limitations of Neurodegenerative Disease Models

Despite significant advances in modeling α-syn and tau-mediated neurodegeneration, no experimental system fully recapitulates the complexity of human disease. Protein overexpression, PFF injection, and genetic models each reproduce distinct aspects of pathology, yet each introduces its own limitations that can complicate interpretation and limit translational relevance.

PFF models have been used to study the prion-like propagation of protein aggregates because they reliably induce templated misfolding and the spread of pathological inclusions [215, 216]. However, these models do not necessarily reproduce the molecular architecture of aggregates found in patients [217]. Recombinant α-syn PFFs generated under different experimental conditions can adopt distinct conformations that differ in seeding efficiency, toxicity, and propagation, resulting in issues with reproducibility [217, 218]. Similar differences have been seen with tau PFFs, with AD brain-derived tau seeding more robustly than recombinant tau PFFs [219]. Consequently, pathology observed following recombinant PFF inoculation may reflect laboratory-specific fibril strains rather than mechanisms underlying human disease [217]. Overall, these limitations mean that PFF models are most useful for dissecting seeded aggregation and spread, but they should not be assumed to fully recapitulate human synucleinopathy or tauopathy biology.

Viral-driven overexpression models provide a rapid means of inducing pathology and are particularly valuable for investigating early mechanisms of cellular dysfunction. However, many viral overexpression techniques rely on α-syn expression that substantially exceeds levels observed in sporadic Parkinson's disease [220]. Moreover, aggregates produced through overexpression may remain confined to transduced neurons and frequently lack the morphology of authentic Lewy bodies [206]. Similarly, tau overexpression models commonly employ high levels of mutant tau to promote rapid neurofibrillary tangle formation, producing pathology on a timescale that differs substantially from the gradual accumulation characteristic of sporadic tauopathies [209]. Viral approaches also introduce potential experimental confounds independent of the transgene itself. In a combined adeno-associated virus (AAV)/PFF model, control animals receiving AAV encoding green fluorescent protein exhibited dopaminergic neuron loss and motor impairments, suggesting that vector delivery or surgical procedures can contribute to neurotoxicity [221].

Genetic models have provided important insights into disease-associated mutations and the long-term consequences of chronic protein dysregulation, yet they also possess inherent limitations. Most α-syn transgenic mice develop widespread protein accumulation and behavioral abnormalities without reproducing the progressive degeneration characteristic of PD [222, 223]. Similarly, many tauopathy models are based on frontotemporal dementia-associated FTDP-17 mutations containing only 4R isoforms of tau, making them highly relevant for inherited tauopathies but less representative of the more common sporadic tauopathies that involve both 3R and 4R tau [224]. In contrast, transgenic mice expressing wild-type human tau often fail to develop robust neurofibrillary pathology [225]. Additionally, transgene insertion sites, copy number, promoter choice, and strain background can all influence the severity and distribution of pathology, making it difficult to distinguish genuine tau and α-syn-mediated effects from model-specific artifacts [223, 225].

Collectively, these considerations emphasize that experimental models should be viewed as complementary rather than comprehensive representations of disease. PFF models are best suited for investigating seeded aggregation and pathological spread, protein overexpression models enable rapid assessment of protein toxicity and region-specific pathology, and genetic models provide insight into mutation-specific mechanisms. Careful selection of experimental systems based on biological questions, combined with validation across multiple model types, is essential to distinguish genuine disease mechanisms from artifacts arising from the model used.

### Conclusions

Collectively, the evidence reviewed here demonstrates that the relationship between tau and α-syn extends far beyond their frequent co-occurrence in Parkinson’s disease. Their interplay is mediated through multiple interconnected mechanisms, including direct protein–protein interactions, reciprocal posttranslational modifications, coaggregation, and convergence on shared pathways involving mitochondrial dysfunction, impaired proteostasis, neuroinflammation, and calcium dysregulation. Rather than representing parallel pathological processes, these mechanisms form a network of self-reinforcing events that promote protein misfolding, neuronal dysfunction, and disease progression. Importantly, this integrated framework provides a mechanistic basis for the greater pathological burden, faster clinical progression, and increased cognitive impairment observed in patients with mixed tau and α-syn pathology.

These findings challenge the traditional view of PD as a purely α-syn-driven disorder and instead support a broader model in which interacting proteinopathies shape disease onset, progression, and phenotypic heterogeneity. While α-syn remains the defining pathological hallmark of PD, tau appears to function as an important modifier of disease severity through reciprocal interactions that amplify cellular dysfunction. More broadly, the extensive overlap between tau- and α-syn-mediated mechanisms underscores the need to move beyond single-protein models of neurodegeneration toward a systems-level framework that considers the impact of multiple pathogenic proteins and cellular stress pathways converging to drive pathology.

This evolving perspective has important implications for both experimental design and therapeutic development. Future studies should prioritize models that more faithfully recapitulate mixed proteinopathies, define the temporal and molecular factors that influence α-syn-tau interactions, and identify biomarkers capable of detecting copathology throughout disease progression. In particular, PD patient-derived organoids offer a promising platform for modeling these complex interactions in a human genetic context, enabling investigation of how pathology develops over time and influences neuronal and glial dysfunction [226, 227]. Combining organoid systems with complementary in vivo models may provide a more comprehensive understanding of disease mechanisms while improving the translational relevance of preclinical findings [228]. Likewise, therapeutic strategies that simultaneously target tau and α-syn, or their shared downstream mechanisms, may prove more effective than approaches directed at either protein alone. Recognizing neurodegenerative diseases as multifactorial disorders driven by interconnected pathogenic networks will be essential for developing disease-modifying therapies capable of addressing the biological complexity and clinical heterogeneity of PD.

## Data Availability

No datasets were generated or analyzed during the current study.
